# Growing Industries, Growing Invasions? The Case of the Argentine Ant in Vineyards of Northern Argentina

**DOI:** 10.3390/insects9010011

**Published:** 2018-01-29

**Authors:** Maria Schulze-Sylvester, José A. Corronca, Carolina I. Paris

**Affiliations:** 1 FCN-IEBI (Instituto para el Estudio de la Biodiversidad de Invertebrados), CONICET CCT-Salta, Facultad de Ciencias Naturales, Universidad Nacional de Salta, Av. Bolivia 5150, CP 4400 Salta, Argentina; jcorronca@gmail.com; 2 Departamento de Ecología, Genética y Evolución, Facultad de Ciencias Exactas y Naturales, Universidad Nacional de Buenos Aires, 1428 Buenos Aires, Argentina; baikibadai@yahoo.com

**Keywords:** *Linepithema humile*, vineyard, invasive species, Argentina

## Abstract

The invasive Argentine ant causes ecological and economic damage worldwide. In 2011, this species was reported in vineyards of Cafayate, a wine-producing town in the Andes, Argentina. While the local xeric climate is unsuitable for Argentine ants, populations could establish in association with vineyards where human activity and irrigation facilitate propagule introduction and survival. In 2013–2014, we combined extensive sampling of the area using ant-baits with monitoring of the change in land use and vineyard cultivated area over the past 15 years. Our results revealed that the species has thus far remained confined to a relatively isolated small area, owing to an effective barrier of dry shrublands surrounding the infested vineyards; yet the recent expansion of vineyard acreage in this region will soon connect this encapsulated area with the rest of the valley. When this happens, vulnerable ecosystems and the main local industry will be put at risk. This case provides a rare opportunity to study early invasion dynamics and reports, to the best of our knowledge, for the first time, the Argentine ant in high altitude agroecosystems.

## 1. Introduction

The Argentine ant, *Linepithema humile* (*L. humile)* (Mayr, 1868), is native to the Paraná River watershed [1], but its invasive range covers parts of Europe, North America, South Africa, Japan, Australia and New Zealand, Hawaii, Easter Island, Azores, Madeira, Gran Canary, and other oceanic islands [2]. *Linepithema humile* is listed as one of the 100 worst invasive species by the International Union for the Conservation of Nature (IUCN) [3]. Soon after colonization of an area, this ant outnumbers native ants and displaces them through aggressive behavior and exploitative and interference competition [4]. *Linepithema humile* also drives changes in the populations of other arthropods (e.g., flies, beetles, spiders, and wasps) which can subsequently lead to impacts on a great variety of other taxa, such as plants, birds, and reptiles [4,5]. Owing to their mutualistic relationship with several honeydew-excreting hemipterans (e.g., scales, aphids, mealybugs), the Argentine ant is considered a pest organism in agriculture. Ants tend hemipterans and provide protection from natural enemies in exchange for honeydew. *L. humile* is associated with mealybug outbreaks in vineyards and orchards worldwide [6,7,8], and can interfere with biological control of mealybugs [9]. Researchers in California, South Africa, and New Zealand recommend control of *L. humile* in order to control mealybugs in vineyards [6,7,9,10].

Dispersal of *L. humile* can take place in two ways: (1) colony budding, when inseminated queens and a group of workers leave the nest on foot and form a new nest nearby (diffusion dispersal); and (2) human-mediated jump dispersal, when humans facilitate the transport of a species into a new range, which has been the dominant pattern in *L. humile* invasions [11]. While Argentine ants depend on high soil moisture levels to survive and spread successfully [12,13], stable populations may persist in areas with unfavorable climates in association with human activity [11]. Vineyards are a particularly suitable habitat for *L. humile* because they can provide abundant food, effective transportation avenues (drip tubes and watering channels), and an overall benign environment fostered by year-round irrigation [14].

**Figure 1:**
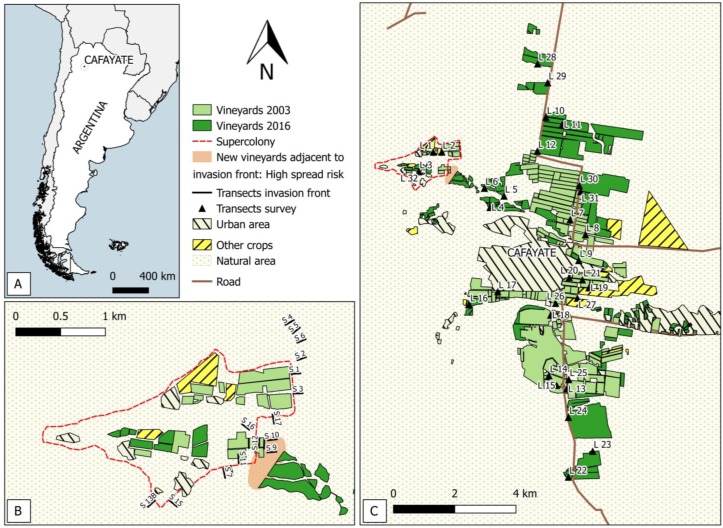
(**A**) Location of Cafayate; (**B**) Limits of the area invaded by *L. humile* (red dashed line, Paris 2011) and sampling transects (S1 to S17) in the invasion front; area with high risk of colony advance is marked in light red; (**C**) Evolution of vineyard land between 2003 (light green) and 2016 (dark green), and location of sampling transects (L1 to L32) for early detection of *L. humile* in other locations within the Cafayate area. Urban areas (grey, hatched), other crops (yellow, hatched), and natural area (grey dots) are displayed as well.

In 2011, *L. humile* was reported in vineyards of Cafayate, a wine producing region in the Andean province of Salta, Argentina, which is approximately 1000 km outside of the *L. humile* native range [15] (Figure 1A). The valley’s temperate desert climate (rainfall <200 mm/year) is ideal for grape and wine production, which is, along with tourism, the most important industry of this rural area. In Cafayate, *L. humile* has formed an isolated supercolony (i.e., a polydomous system lacking intraspecific aggression) that, so far, has spread over approximately 300 ha of vineyards [15] (Figure 1B,C). These infested vineyards are situated in Yacochuya, 6 km northwest of the town of Cafayate. The site is at approximately 2000 m above sea level, effectively isolating these vineyards from the larger wine producing valley, which is located at a lower elevation and separated by a large expanse of natural habitat (i.e., dry-bush). Typical plants of these shrublands are *Cercidium praecox*, *Echinopsis atacamensis*, *Prosopis ferox*, *Acacia caven*, *Senna* sp., *Opuntia ficus*, etc. However, recent expansion of planted vineyard acreage in this region threatens to remove this natural barrier between the infested vineyards and the main production areas of the valley. The spread of *L. humile* into other neighboring farms and from there to the rest of the valley presently constitutes a real risk.

Vine mealybug, *Planococcus ficus* (Singoret, 1875) is a principal pest of vineyards in the Cafayate region [16]. According to the National Institute of Agricultural Technology (INTA), *P. ficus* was first detected in Cafayate in 2001 and is usually managed with pesticides, although mating disruption with pheromone dispensers has been shown to be more effective. *P. ficus* is almost exclusively found in vineyards, while local orchards (peach, quince, and plums) remain uninfested; sporadically, it has been detected in ornamental plants and fig trees in private homes (personal communication). As mentioned above, infestations of *P. ficus* can be intensified in the presence of *L. humile*. Thus, further expansion of *L. humile* into the Cafayate region would likely exacerbate *P. ficus* outbreaks and lead to negative impacts on production. Furthermore, expansion of *L. humile* populations in this region will likely have impacts in non-crop, natural habitats such as the adjacent Reserva Natural Quebrada de las Conchas. While other natural habitats in the area are too dry for *L. humile*, this natural reserve includes the region’s only permanent river (Río de las Conchas) which provides possibly suitable habitats for *L. humile*.

To our knowledge, the Argentine ant population in Cafayate is the only one reported in high altitude agroecosystems. While Argentine ants are considered a pest in many wine producing areas around the world [6,8,10], the only report from comparable elevations origins from a national park in Hawaii [17] whose cold and humid climate conditions differ substantially from the ones found in Cafayate and most wine producing areas.

Invasive species are causing great economic damage in many developing countries, where agriculture is the main engine for the economy [18]. Studying invasive species in these systems can help developing countries to protect their most valuable resources and provide measures against threats posed to their economy by these organisms. However, there is little research on insect invasions in South America [19,20] and in high elevation agroecosystems [21].

It has been suggested that extreme climate conditions, high altitude, and remoteness are factors that lower the invasibility of an ecosystem [12,22,23]. Yet, *L. humile* has been reported in high altitude locations which appeared to be isolated from the invaded range [15,17]. Despite the altitude, remoteness, and steep environmental gradients, agriculture and human populations are common in tropical and subtropical mountains [24,25]. Human activity is closely related to factors that facilitate invasions, such as the dispersal of propagules and disturbance of the environment [26]. As a result, these habitats are not necessarily immune to biological invasions. In the present study we investigate the following questions: (1) Is the Cafayate *L. humile* supercolony encapsulated? (2) Which factors put this encapsulation at risk and facilitate a spread? (3) Are there other populations of *L. humile* in the area? We conduct intensive sampling to assess the evolution of the invasion front and surveyed the area around Cafayate for other *L. humile* colonies. This information is used in combination with quantitative data on the evolution of the spatial distribution of vineyard land in the area over the last 15 years to assess the species expansion risk.

## 2. Materials and Methods

We obtained official data on vineyard surface for the years 2001 and 2015 from the Argentine National Viticulture Institute (Instituto Nacional de Viticultura, INV). We complemented this information with satellite pictures from Google Earth (http://www.google.com/earth/index.html) processed with QGIS (an open source geographic information system) [27]. Thus, pictures from 2003 were compared with pictures from 2016 to assess vineyard surface change around Cafayate during this period. In areas where land use could not be satisfactorily resolved from these images, we conducted field checks. In this way, we obtained a complete and accurate picture of the vineyard distribution that allowed us to assess the evolution of the distance between invaded and non-invaded vineyard patches in the Cafayate area over the period studied.

Between February 2013 and February 2014, we studied the fluctuations of the eastern border of the invasion front of the *L. humile* colony in Cafayate using bait transects. We established 17 (2013) and 16 (2014) 100 m transects perpendicular to the colony border facing towards the valley and neighboring vineyards (Figure 1B), where *L. humile* might find suitable habitats. Baits consisted of 5 mL tubes filled with cotton balls soaked in 25% sugar solution [28,29]. Each transect had 11 equidistant baits that were allowed for ant colonization for 30 min. In 2014, it was not possible to sample transect S16 due to rainfall. Transect S13A to S13B were slightly relocated in 2014 because of their close proximity with other transects.

In May 2014, we conducted an extensive survey for satellite populations of *L. humile* within a radius of 10 km from the town of Cafayate (Figure 1C). Our search focused on habitats such as vineyards, campsites, water reservoirs, and channels, considered suitable for *L. humile* due to their urban/agricultural character. Baits (see above) were laid out at 20 m intervals along 32,200 m, L-shaped transects. Since the aim of this survey was detection, ants were allowed 1 h for colonization of baits. Vineyards in Cafayate are not open to the public. Not all landowners gave permission to enter their properties, and hence some areas could not be sampled.

All sampling of *L. humile* (February 2013, February 2014 and May 2014) was conducted at temperatures between 20 °C and 30 °C, which is optimal foraging temperature for this species [11,30]. Ants from all surveys were fixated in 96% ethanol in situ and stored until final identification. In the laboratory, ants were identified to genus or species (ants belonging to the genus *Linepithema*) level and counted under a stereoscope microscope (maximum magnification 57.5×).

## 3. Results

### 3.1. Vineyard Surface

The area cultivated with vineyards almost doubled during the study period. According to INV, the productive vineyard surface area increased from 1271.89 ha in 2001 to 2318.26 ha in 2015 (82% increase). These figures are in accordance with our observations in situ and using satellite pictures, which yielded an increase of 88.6% over a similar period (from 1113.11 ha of vineyards in 2003 to 2099.3 ha in 2016). In most cases, new vineyard plantings went into areas that were previously natural habitat rather than replacing crops. The increase mostly affected the northern section of the town (65.4% of the new vineyard surface area), while vineyard expansion rates were lower in the southern section (34.6%). As a result, the overall distribution of vineyard land shifted from a situation where up to two-thirds of all vineyards were situated to the south of Cafayate in 2003 into the present situation where vineyards are equally distributed at the northern and southern sides of the town (Figure 1C). Of special interest to our study are new vineyards planted to the northwest of the town in 2015–2016, as they might bridge the invaded and the non-invaded zones of the valley. These vineyards have dramatically diminished the distance between both zones from ca. 2500 m in 2003 to only 250 m today.

### 3.2. Spatio-Temporal Development of the Invasion Front

In 2013, we detected *L. humile* in 6 of the 17 transects sampled, while in 2014 *L. humile* was present in only 4 of 16 transects (Figure 1B, Table 1). *Linepithema humile* was detected in both years only in transects S1 and S2 (Table 1). While the species was found in transects S8, S9, S11, and S16 in 2013, it was not found again at the same sites a year later. In 2014, Argentine ants were only detected in transects S1, S2, S3, and S14, principally in the first few baits towards the invaded area.

### 3.3. Survey of L. humile around Cafayate

A total of 320 samples were collected from 32 sites around Cafayate. Ants were caught in all 32 transects. *Linepithema humile* was detected only within the known invaded area (four transects). Despite the extensive search in the surroundings of Cafayate, we did not discover this species anywhere else. While *L. humile* accounted for the highest abundances in our samples and almost half of all collected ants, the species was only found in 7% of the baits and in 13% of all transects (Table 2).

The genus *Linepithema* was represented only by the species *L. humile*; ants from other genera were not identified to species level and are referred to as ‘other ants’ (Table 2).

## 4. Discussion

This work reports, for the first time to a broad audience (see [15]), a range expansion of the Argentine ant into non-native areas of the province of Salta in northwest Argentina; and reveals that while this introduction is so far confined to a small area, there is a substantial risk for a wide expansion of the species in the near future in association with the rise of the wine industry in the area.

Overall, our data show that the invasion front did not advance but was confined or even receded from its historic limits during the period studied. Our results suggest that the introduction of *L. humile* to Cafayate was restricted to a single area described by Paris [15], from where the species has not spread neither by natural diffusion nor jump dispersal into neighboring areas. This is most probably because the invaded area has historically been isolated by surrounding desert-like lands covered with scant shrub vegetation unsuitable for the Argentine ant. This has drastically changed during the last decade or so, when vineyard area subjected to year-round irrigation has almost doubled, also doubling the potentially invasible area for *L. humile* in Cafayate. By the end of 2016, invaded and non-invaded but suitable habitats were only 250 m apart. As water for vineyard irrigation is usually provided from reservoirs and uphill streams, channels and tubes can easily form corridors for a natural downhill diffusion dispersal of the ant from the elevated fields where it is currently encapsulated towards more connected vineyards in the entire valley.

Our results support the idea that invasibility has to be assessed in view of the characteristics of the transport vector. While Yacochuya, the gateway of the *L. humile* introduction in Cafayate, is geographically remote and with poor connectivity (non-paved roads, little transportation), the presence of viticulture in the area enhances dramatically the potential of propagule transport. The importance of hostile environmental conditions (low humidity, low vegetation cover) is diminished as *L. humile* finds suitable habitats in vineyards and sufficient transport vectors. Pauchard et al. [22] found that the improved infrastructure of mountains (e.g., for tourism) results in increased land use and increased propagule pressure from non-indigenous species which may promote the dispersal of non-indigenous species at a local, regional, and global scale. Cafayate’s blossoming wine industry is evidenced by increased wine tourism—between 2006 and 2013 wineries reported a 30% annual increase in visitors and a 400% increase in tourists traveling the ‘Ruta del Vino’ around Salta [31]. This may transform a remote area into a well-connected local hub, which could result in increased potential for the introduction of invasive species such as *L. humile*. The degree of vector traffic may soon result in a number of new satellite populations [32], especially considering that Argentine ants establish new colonies with as little as 1 queen and 10 workers [33].

The authors have already informed managers (the regional experimental station with the National Institute of Agricultural Technology—INTA) and given public talks and interviews on the local TV and radio with the hope that action may be taken in time.

## 5. Conclusions

We conclude that, for now, the invaded area remains encapsulated. The reported supercolony did not advance, neither by natural colony diffusion nor by jump dispersal. No other populations of *L. humile* were detected in the area around Cafayate. Nevertheless, there is a great risk of spread of *L. humile* towards to rest of the valley due to the recent expansion of irrigated vineyards adjacent to the invaded area. We will continue to monitor the invasion front and the Cafayate area in the coming years. Information on early invasion stages of this global invader is important for ant invasion ecologists worldwide, even more so as this invasion is taking place in the neighborhood of usually understudied native ranges. From the management perspective, an invasion throughout the valley would not only put at risk one of the most important regional industries and impair the local arthropod community, but also increase the chances for jump dispersal into other nearby wine regions. Considering the numerous human activities that could transport viable populations of ants (e.g., exchange of soil, machinery, vines and grapes between wineries, transport of fire and construction wood by the local population), a human-mediated spread of the species in and around Cafayate is only a matter of time, unless immediate measures are taken. These measures should include raising local awareness among local residents and wine grape growers, who do not currently consider this ant to be a significant threat to vineyard production.

## Figures and Tables

**Table 1:** Spatio-temporal development of the invasion front. Transects that detected *L. humile* in 2013 and/or 2014 using 11 traps perpendicular to the invasion front. Trap 1 is closest to the border of the invasion front. Filled squares, traps with *L. humile* (number) present; open squares, traps without *L. humile* present.

Transect	Year	Trap 1	Trap 2	Trap 3	Trap 4	Trap 5	Trap 6	Trap 7	Trap 8	Trap 9	Trap 10	Trap 11
S1	2013		11	20	3			1			21	10
	2014	2			2	1						
S2	2013	1	2	37		2		3	10			3
	2014	16	24		17		4	3				
S3	2013											
	2014	22	9									
S8	2013	1										
	2014											
S9	2013										2	
	2014											
S11	2013						1	2				
	2014											
S14	2013											
	2014	1	2									
S16	2013		23			48	9	17	31			
	2014	NA ^1^	NA ^1^	NA ^1^	NA ^1^	NA ^1^	NA ^1^	NA ^1^	NA ^1^	NA ^1^	NA ^1^	NA ^1^

^1^ Transect not sampled due to rainfall.

**Table 2:** Number of ants sampled using bait transects in the Cafayate area in 2014. Total number of individual, baits, and transects indicated.

Genus/Species	Caught Individuals		Samples (Baits)		Transects	
	Number	Percent (%)	Number	Percent (%)	Number	Percent (%)
*Linepithema humile*	941	49.79	23	7.19	4	12.50
Other ants	949	50.21	164	51.25	31	96.88
Total	1890	-	320	-	32	-
